# Evaluating Biochar’s Role in Dye Adsorption and Wheat Performance Under Saline Conditions

**DOI:** 10.3390/ma18204678

**Published:** 2025-10-12

**Authors:** Ghenwa Kataya, Dalia El Badan, David Cornu, Assi Al Mousawi, Mikhael Bechelany, Akram Hijazi

**Affiliations:** 1 Research Platform for Environmental Science (PRASE), Doctoral School of Science and Technology, Lebanese University, Hadath 1519, Lebanon; ghenwa.kataya.1@ul.edu.lb (G.K.); akram.hijazi@ul.edu.lb (A.H.); 2 Institut Européen des Membranes, IEM–UMR 5635, University of Montpellier, CNRS, ENSCM, Place Eugène Bataillon, 34095 Montpellier, France; david.cornu@umontpellier.fr; 3 Department of Biological Sciences, Faculty of Science, Beirut Arab University, Beirut P.O. Box 11-5020, Lebanon; d.badan@bau.edu.lb; 4 Botany and Microbiology Department, Faculty of Science, Alexandria University, Alexandria 21568, Egypt; 5 Department of Biological and Chemical Sciences, School of Arts and Sciences, Lebanese International University, Khiyara—West Bekaa, Beirut P.O. Box 146404, Lebanon; assi.mousawi@liu.edu.lb

**Keywords:** biochar, valorization, salinity, dye, crystal violet, cheat

## Abstract

This research explores the dual role of biochar in addressing the escalating challenges of water salinity and pollution, focusing on its potential for both wastewater treatment and agricultural resilience. We investigated the adsorption capacity and efficiency of various biochar treatments to remove crystal violet dye from contaminated water. Biochar treated with H_2_SO_4_ demonstrated the highest adsorption capacity (450 mg/g). It consistently achieved 100% removal efficiency in all crystal violet concentrations tested, while silver-modified biochar showed a 99.95% removal rate at 50 ppm and an adsorption capacity of 5 mg/g. In agricultural applications, we evaluated the impact of biochar applications at concentrations of 1% and 3% on wheat crops irrigated with saline water of varying conductivity levels (0.63 and 10 dS/m). Wheat plants treated with 1% biochar exhibited the highest yield (26.6 cm) under 0.63 dS/m water conductivity, significantly outperforming the control group (17 cm). Biochar also resulted in elevated chlorophyll levels, with chlorophyll a ranging from 29.8 to 20.9 µg/mL and chlorophyll b ranging from 54 to 23 µg/mL, showing a marked improvement over the control. These findings demonstrate biochar’s potential to mitigate salinity-induced damage, with lower salinity conditions further enhancing chlorophyll a levels, while untreated plants showed reduced chlorophyll under high salinity.

## 1. Introduction

Climate change reduces freshwater availability and increases the risk of water pollution, making the protection of existing water sources essential [1,2]. Untreated wastewater is a major source of pollution, containing hazardous substances such as industrial chemicals, microorganisms, and persistent, toxic, and non-biodegradable dyes [3,4]. Crystal violet (CV), a widely used synthetic dye, is particularly concerning, as it is mutagenic, carcinogenic, and resistant to degradation [5,6].

Various wastewater treatment methods exist, including solvent extraction, ion exchange, photocatalysis, nanofiltration, chemical precipitation, and electrochemical treatment, with adsorption preferred for its cost-effectiveness, efficiency, and simplicity [7,8,9,10,11]. Biochar, a carbonaceous material produced from the pyrolysis of organic biomass under limited oxygen, has emerged as a sustainable adsorbent for wastewater treatment [12,13,14]. Its large surface area, porosity, and multiple functional groups enable efficient dye removal [15,16]. Furthermore, biochar production from biomass waste offers an eco-friendly and sustainable solution for wastewater treatment, reducing environmental pollution and contributing to carbon sequestration [17,18,19].

Biochar removes dyes through multiple mechanisms, including electrostatic attraction between dye cations and negatively charged functional groups, π–π stacking with aromatic structures, hydrogen bonding, and pore-filling effects, with previous studies confirming its effectiveness in adsorbing crystal violet via physical adsorption, chemical interactions, and ion exchange [20,21,22]. However, the application of biochar produced from kitchen waste for CV removal has not been extensively explored.

Another critical challenge that affects water and soil quality is salinity. Soil salinity, particularly the accumulation of Na^+^ ions, reduces plant water uptake and nutrient availability, ultimately leading to yield loss [23,24]. Biochar can alleviate these effects by improving cation exchange capacity, replacing Na^+^ with Ca^2+^ and Mg^2+^, enhancing osmotic balance, and providing habitats for salt-tolerant microbes that aid in soil rehabilitation [25].

Biochar has demonstrated remarkable potential to alleviate the adverse impact of soil salinity, as results have shown its superior adsorption performance and ability to improve soil properties. Studies have demonstrated that the application of biochar in saline soils improves the productivity of crops and efficiency of water use. Biochar has shown potential to mitigate soil salinity by improving soil properties and crop performance. For instance, Zhang et al. (2020) [26] reported that biochar increased soybean yield by 3.1–14.8% under salinity stress and improved efficiency of water use by 15.6%. Similarly, Raziye et al. (2021) [27] showed that biochar application at 5% and 10% enhanced tomato plant height by 43.1%, leaf area by 45.3%, and chlorophyll-a content under saline conditions. These results highlight biochar’s effectiveness in enhancing crop growth and resilience in saline soils.

Importantly, the challenges of wastewater contamination and soil salinity are interlinked within the broader framework of sustainable water management and agricultural resilience. In many regions, treated or untreated wastewater is reused for irrigation due to scarcity of freshwater, but its pollutant load, including toxic dyes, can worsen soil quality and exacerbate salinity stress over time [28]. Thus, developing solutions that simultaneously purify contaminated water and ameliorate saline soils is crucial to closing the loop between water and agriculture. Biochar, with its dual ability to absorb pollutants from wastewater and rehabilitate saline soils, represents a unique material to bridge these challenges.

This study aims to explore the dual role of biochar produced from kitchen waste in addressing these interconnected issues. Specifically, the performance of biochar derived from kitchen waste in eliminating CV from contaminated water was investigated. Biochar will be tested in both its activated and modified forms, with various factors influencing its performance while being assessed. Additionally, we evaluate the ability of biochar to mitigate the negative impact of saline irrigation on wheat growth and yield. Biochar at concentrations of 1% and 3% was applied as soil amendments and the length of the wheat crop and the chlorophyll a and b content were measured. The objective of this study is to provide insights into the potential of waste-derived biochar as a sustainable, circular solution for wastewater treatment and for enhancing agricultural resilience in saline environments.

## 2. Materials and Methods

### 2.1. Biochar Preparation

Biochar was produced from a mixture of orange peels, coffee residue, banana peels, and potato peels by pyrolyzing at 400 °C for 1 h in a muffle furnace(DAIHAN, WiseTherm FP, Seoul, Republic of Korea). The biochar preparation method was determined through earlier optimization studies, following the method of Kataya et al. (2024) [29]. The activated biochar was then produced by treating the biochar with concentrated sulfuric acid (H_2_SO_4,_ 95%,UNI-CEM Pvt. Ltd., Punjab, India) in a 1:3 ratio, heating at 200 °C for 1 h, and cooling to room temperature. The resulting slurry was dispersed in ultrapure water, and the solid residue was filtered and washed until the effluent reached a neutral pH, as described by Peixoto et al. (2021) [30].

### 2.2. Crystal Violet Removal

In this section, untreated biochar, activated biochar, Ag-Biochar composite, and magnetized biochar were prepared to evaluate their effectiveness in removing CV and to compare their performance. For the Ag-Biochar composite, 10 g of sodium hydroxide (NaOH, pellets, 98% purity, Alfa Aesar GmbH & Co., Karlsruhe, Germany) was dissolved in 100 mL of ultrapure water, stirred, and sonicated for 20 min. Then, 100 mL of ultrapure water and 3 g of silver nitrate (AgNO_3_, molecular weight 169.88 g/mol, purity 99.8%, UNI-CEM Pvt. Ltd., Punjab, India) were added and sonicated for another 20 min. The pH was adjusted to 14.5 with NaOH solution, stirred and filtered to obtain silver hydroxide powder. Separately, 100 g of feedstock powder was mixed with 500 mL of alcohol and stirred for 3 h. Silver hydroxide was added to this solution, stirred for 24 h and heated to 60 °C to remove the alcohol, leaving solid material. This solid was then pyrolyzed at 400 °C for 1 h, following Chen et al. (2019) [31]. Magnetized biochar was prepared by dissolving FeCl_3_·6H_2_O (1.80 g) and FeSO_4_·7H_2_O (1.85 g)(Reagent grade, purity 98% and 99% respectively, Sigma-Aldrich, Steinheim am Albuch, Germany) in 150 mL of distilled water, heating to 60 °C for 5 min, and adding 2.0 g of biochar to the solution. This mixture was stirred for 30 min, and NaOH was added until the pH reached 11, changing the suspension color from brown to black. The mixture was then heated to 80 °C for 1 h and aged at room temperature for 2 h. Magnetized biochar was separated using a magnet, washed with distilled water and ethanol, filtered, and dried at 50 °C in a vacuum drying chamber, following Mohan (2014) [32] and Sun et al. (2022) [33].

### 2.3. Batch Adsorption Experiment

Batch adsorption experiments were carried out in 50 mL glass bottles under ambient temperature and atmospheric pressure. Each experiment was conducted in triplicate, and the mean values were reported. Blank samples were included for quality assurance purposes. A stock solution of Crystal Violet (CV) dye (1000 mg/L) was prepared by dissolving 1 g of CV in ultrapure water. Several parameters were investigated to assess their influence on the adsorption capacity of the biochar. All chemicals used in this study were of analytical grade and high purity. CV dye (molecular formula: C_25_H_30_N_3_Cl; molecular weight: 407.98 g/mol) was obtained from HIMEDIA, India, Mumbai. After adsorption, the mixtures were filtered, and the absorbance of the remaining CV solution was measured using a UV–Vis spectrophotometer (Hitachi, Tokyo, Japan) at the maximum wavelength of 583 nm. We investigated the effect of initial CV concentrations ranging from 10 to 50 ppm and 0.5 g of biochar (activated or non-activated) with a contact time of 1 h. All adsorption experiments were performed at ambient room temperature (25 °C) under atmospheric pressure. The removal efficiency of biochar was compared between activated and non-activated biochar, with activation performed using sulfuric acid. Additionally, various modified biochar forms (Ag-Biochar, Fe-Biochar, and H_2_SO_4_-Biochar) were evaluated for their effectiveness in CV removal. The samples were filtered using a syringe PTFE membrane filter and analyzed using UV-spectroscopy to measure the absorbance of the solution.

The equilibrium adsorption of CV on the adsorbent was quantified based on Equation (1), as presented below:

(1) $$\mathrm{qe}=\;\frac{(C0-Ce)\;\;\;}{\;m}\times \;v$$

where $q_{e}$ represents the equilibrium adsorption capacity (mg/g); $C_{0}$ and $C_{e}$ denote the initial and equilibrium concentrations of CV in the solution (ppm), respectively; V is the volume of the solution (L); and m is the mass of the biochar (g). The removal efficiency (R) was calculated using the following Equation (2):

(2) $$R\;=\frac{\;\;\;(C0-Ce\;)\;\;}{C0}\times \;100$$

### 2.4. Biochar Characterization

X-ray photoelectron spectroscopy (XPS) was applied to analyze the elemental composition of modified biochar samples, verifying the successful modifications. The analyses were performed using an ESCALAB 250 (Thermo Electron, ThermoFisher, Waltham, MA, USA) with a monochromatic Al Kα excitation source (1486.6 eV). The analyzed surface had a diameter of 500 μm, and the photoelectron spectra were adjusted against the C-C component of the C1s carbon peak at 284.8 eV. Charging effects were mitigated using a low-energy electron beam (−2 eV).

Brunauer–Emmett–Teller (BET) analysis was conducted to determine the specific surface area and pore size distribution of the biochar samples. Measurements were carried out using a 3Flex instrument (Micromeritics) with N_2_ as the adsorptive gas. Before analysis, samples were degassed at 200 °C overnight to remove any surface contaminants.

### 2.5. Adsorption Isotherm

Adsorption isotherms were employed to describe the relationship between the adsorption capacity and the equilibrium concentration of the adsorbate. The Langmuir and Freundlich models were applied to analyze the isotherm parameters using the corresponding Equations (3) and (4):

(3) $$\frac{Ce\;}{qe\;}\;=\;\frac{1}{KL\;\times \;qm\;}+\;\frac{Ce}{qm\;}$$

(4) $$\ln qe=\frac{1}{n}\ln Ce-\ln kf$$

where $C_{e}$ and $q_{e}$ represent the equilibrium concentration of CV in the solution (mg/L) and the corresponding equilibrium adsorption capacity (mg/g), respectively; $q_{m}$ denotes the maximum adsorption capacity of CV (mg/g); $K_{L}$ is the Langmuir constant (L/mg); and $K_{F}$ represents the Freundlich constant (L/mg).

### 2.6. Soil Amendment and Irrigation Experiment

An experiment was conducted to assess the effects of biochar as a soil amendment on wheat plant growth and yield in Riyyak soil (33.870399°, 35.965320°), collected from the Beqaa Valley of Lebanon, and it is clayey in texture with a slightly alkaline pH of 7.7, low organic matter (1.56%) and organic nitrogen (0.09%), moderate electrical conductivity (0.27 ms/cm), low carbonate content, and relatively high available phosphorus (94 ppm). It represents typical agricultural soil suitable for assessing the effects of salinity and biochar amendments under irrigation with low and high-salinity water. Ten wheat seeds were sown per pot and maintained at 25 °C under 16 h light conditions for two weeks. Following the methodology of Yerli (2023) [34], two concentrations of biochar (1% and 3%) were applied as amendments, alongside a control group without biochar. Each treatment was applied in four independent replicates (n = 4) in a completely randomized design. After allowing the wheat seeds to germinate and grow for two weeks, saline solutions were prepared by dissolving sodium chloride in distilled water. Two concentrations were used: 10 ds/m and 0.63 ds/m. After the growth period, the wheat plants were irrigated with saline solutions every two days. Subsequently, crop length and chlorophyll content were measured to evaluate plant growth and performance after harvest.

Data were subjected to one-way ANOVA to evaluate the effects of biochar dosage and irrigation water salinity on wheat growth parameters. When significant differences were observed, Tukey’s Honestly Significant Difference (HSD) test was used for pairwise comparisons of treatment means. All statistical analyses were conducted using SPSS software (version 23), and differences were considered statistically significant at p<0.05.

## 3. Results

This section consists of three major parts: (1) the characteristics of biochar, (2) the removal of crystal violet dye under various influencing factors, and (3) the usage of biochar as a soil amendment, presenting its effectiveness in mitigating salinity stress and its impact on wheat growth and chlorophyll content.

### 3.1. Biochar Characteristics

The XPS analysis demonstrated valuable insights into the successful modifications of biochar, confirming the incorporation of specific elements that reflect the effectiveness of each modification (Table 1). For Biochar-Fe, the XPS analysis showed the presence of iron (Fe) at 18.1%, which is a clear indication of a successful modification with iron (Table 1a). In the case of Biochar-Ag, the presence of silver (Ag) at 0.2% confirmed the successful integration of silver nanoparticles onto the surface of biochar (Table 1b). Finally, for Biochar-H_2_SO_4_, the XPS results revealed a sulfur (S) content of 3%, which directly indicates the successful modification of biochar through sulfuric acid treatment (Table 1c).

The results of BET represented in Table 2 indicate that Biochar-H_2_SO_4_ exhibited the highest BET surface area at 33.0 m^2^/g, significantly outperforming the other types of biochar. In comparison, the surface area of Biochar-Fe was 4.76 m^2^/g, which is slightly higher than that of Biochar-Ag (3.4 m^2^/g), but still lower than that of non-modified Biochar (4.8 m^2^/g). The substantial increase in surface area observed in Biochar-H_2_SO_4_ can be linked to the acid activation process (H_2_SO_4_), which likely improved its porosity and surface reactivity. The lower surface areas of Biochar-Fe and Biochar-Ag may be linked to the presence of metal-based modifications, which may alter the surface chemistry and structure without significantly increasing the porosity.

### 3.2. Crystal Violet Removal

**Figure 1:**
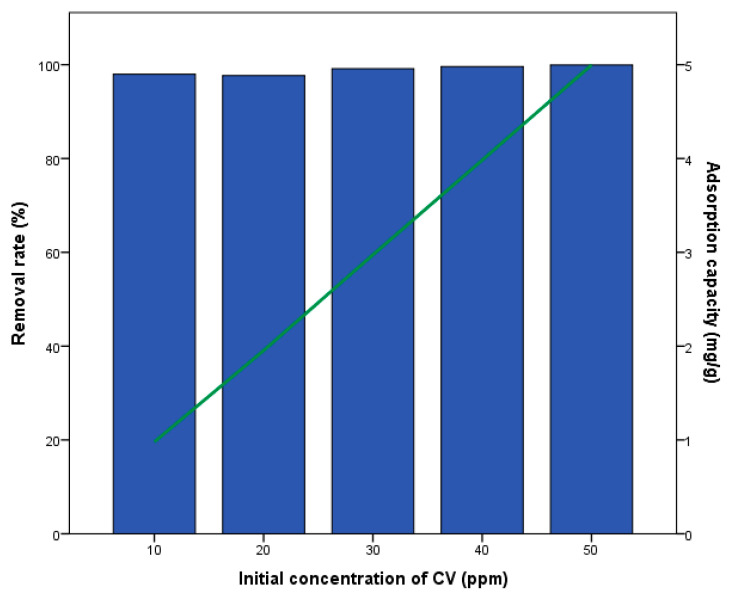
Effect of biochar loaded with silver nitrate on the removal rate and adsorption capacity of CV.

The adsorption process reached its highest efficiency (99.95%) at a 50 ppm initial concentration, with a maximum adsorption capacity of 5 mg/g (Figure 1). The initial concentration of silver biochar significantly influences the adsorption rate and adsorption capacity of CV. The results indicated that increasing the concentration of silver biochar enhances both the removal rate and the adsorption capacity. Our findings are consistent with those of the literature, which consistently demonstrate the effectiveness of silver-based biochar in adsorbing various organic pollutants. Akram et al. (2021) [35] indicated a rapid increase in the removal percentage with increasing adsorbate concentration for dyes such as rhodamine B and Congo red, aligning with our observed adsorption mechanism. The rapid increase in the removal percentage with increasing adsorbate concentration and subsequent saturation at higher concentrations indicates the availability of numerous active binding sites on the silver biochar surface. This rapid initial adsorption, followed by a plateau, underscores the efficiency of silver biochar in dye removal applications. Similarly, Tran et al. (2021) [36] showed a significant improvement in the adsorption efficiency of silver-modified biochar composites for dyes like methylene blue, methyl orange, and rhodamine B with increasing concentration and contact time. In addition, studies on wheat straw biochar composites with silver nitrate by Khan et al. (2022) [37] have shown enhanced adsorption compared to biochar alone. This suggests that the integration of silver nanoparticles into biochar matrices significantly improves the adsorption capabilities, likely as a result of the enhanced surface area and accessibility of active sites for pollutant binding. The integration of silver biochar proves to be highly effective in the adsorption and removal of CV and other organic dyes. The increased initial concentration of silver biochar leads to higher removal rates and adsorption capacities, corroborating the enhanced performance observed in the literature for similar silver-modified biochar composites. This enhancement can be due to the increase in the number of active binding sites and the synergistic effects between the silver nanoparticles and the biochar matrix. These observations demonstrate the potential of silver biochar as a promising adsorbent for wastewater treatment applications. The comparison reveals that silver nitrate-modified biochar outperforms non-activated, non-modified biochar in terms of both removal rate and adsorption capacity for CV. The improved efficiency of the modified biochar can be attributed to the presence of silver nanoparticles, which are likely to increase the surface area and the number of active sites available for adsorption. This modification improves the overall efficiency of the biochar, making it a more effective adsorbent for the removal of organic pollutants like CV from aqueous solutions. Moreover, while silver nanoparticles (Ag-NPs) are effective catalysts for reducing organic dyes, they are prone to oxidation and agglomeration during catalysis. To address this issue, additional support, such as embedding Ag-NPs in biopolymer composites like biochar, has been introduced. This enhancement improves the stability and effectiveness of Ag-NPs, and numerous studies are currently exploring the use of silver nanocomposites to remove toxic dyes and pollutants from water [38].

**Figure 2:**
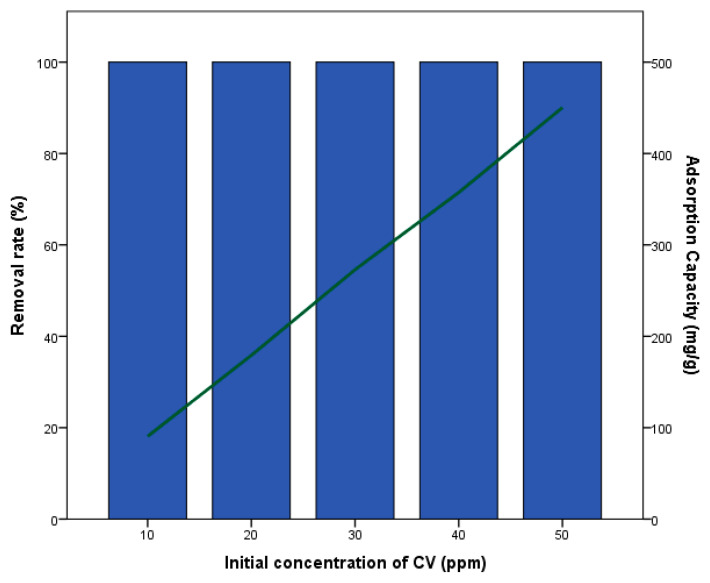
Effect of initial concentration on the removal rate and adsorption capacity of H_2_SO_4_-activated biochar.

Figure 2 shows the effect of initial concentration on CV removal rate and adsorption capacity by activated biochar. H_2_SO_4_-treated biochar showed the highest improvement in removal percentage. The results indicate that the removal rate remained constant at 100%, while the adsorption capacity increased with the initial concentration, ranging from 90 to 450 mg/g. This suggests that the adsorption capacity of the H_2_SO_4_-treated biochar is highly effective in removing CV, with activation enhancing the efficiency by 107.14 times compared to the non-activated biochar. Moreover, the enhanced adsorption capacity of H_2_SO_4_-modified biochar can be attributed to its higher oxygen content (24.2%) as revealed by XPS analysis, which indicates abundant –OH and –COOH groups capable of interacting with crystal violet molecules. Additionally, the BET surface area of 33.0 m^2^/g provides more accessible adsorption sites compared to metal-modified biochars. The combination of increased surface area and functional group availability explains the superior adsorption performance of Biochar-H_2_SO_4_, whereas Biochar-Fe and Biochar-Ag exhibit lower capacities due to reduced surface area and fewer oxygenated functional groups. Comparing our results to the literature, Baharim et al. (2023) [39] reported that biochar prepared at 300 °C from banana pseudo stem (BPS) waste and activated by H_2_SO_4_ showed an adsorption capacity of 9.8 mg/g and a removal rate of approximately 98.4%. The obtained results in this study surpass these findings, indicating the superior performance of H_2_SO_4_-treated biochar. Additionally, Mohanty et al. (2006) [40] found that the maximum uptakes of CV by sulfuric acid-activated and zinc chloride-activated rice husk biochar prepared at 300 °C were 64.875 and 61.575 mg/g, respectively. These values are significantly lower than the adsorption capacity observed in our study. Furthermore, Chen et al. (2010) [41] reported that *Phragmites australis* biochar activated by H_3_PO_4_ and prepared at 450 °C had an adsorption capacity of approximately 476.19 mg/g, which is comparable to our results. Overall, these findings suggest that H_2_SO_4_-treated biochar is highly effective for CV removal, with significant improvements in adsorption capacity.

**Figure 3:**
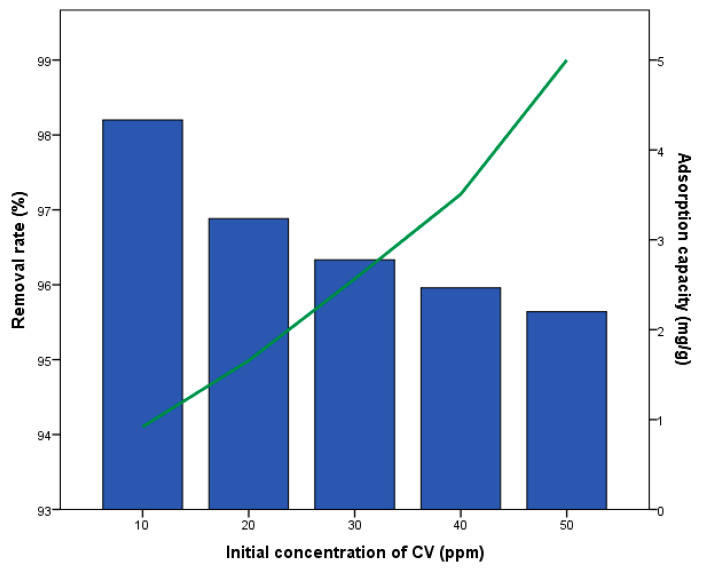
Effect of initial CV concentration on the removal rate and the adsorption capacity of CV by biochar (non-modified).

Figure 3 illustrates the effect of the initial concentration of CV on the removal rate and adsorption capacity of non-activated, non-modified biochar. The removal rate ranged from 95.6% to 98.2%, while the adsorption capacity varied between 0.9 and 4.2 mg/g. As the initial concentration of CV increased, the removal rate decreased, whereas the adsorption capacity increased. This phenomenon was reported by Nouioua et al. (2023) [23] and Sun et al. (2023) [42]. At lower CV concentrations, there is a significant concentration difference between the solution and the biochar surface. This difference causes more CV molecules to move from the solution to the biochar surface, filling up the active sites available for adsorption. However, as the CV concentration continues to rise, the biochar approaches its maximum adsorption capacity. Once the biochar is saturated, it cannot adsorb any more CV, leading to an increase in the amount of CV left in the solution and a corresponding decrease in the removal rate, as explained by Sun et al. (2023) [42].

**Figure 4:**
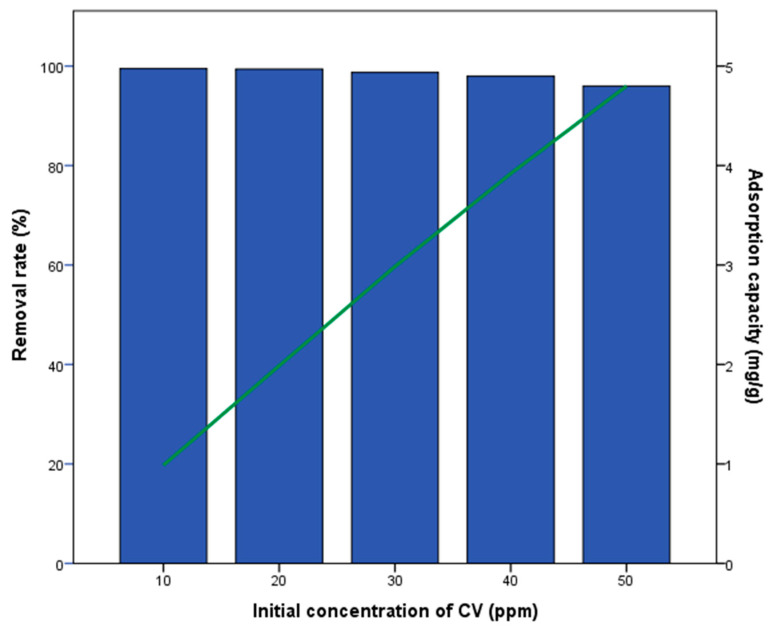
Effect of the initial CV concentration on the removal rate and CV adsorption capacity by magnetized biochar.

Figure 4 presents the influence of the initial CV concentration on the adsorption capacity and removal efficiency of magnetized biochar. The removal efficiency varied from 96% to 99.5%, while the adsorption capacity ranged from 0.98 to 4.7 mg/g. As the initial CV concentration increased, the removal efficiency slightly declined, whereas the adsorption capacity showed an increasing trend. Overall, the results indicate that magnetized biochar exhibits slightly higher adsorption performance compared to non-modified biochar. However, its advantage remains in facilitating biochar removal after water treatment due to the incorporated magnetic properties. Also, Kumbhar et al. (2022) [43] have found a similar removal rate ratio of approximately 98.7% for magnetized biochar. Luyen et al. (2019) [44] reported that magnetized biochar had better results compared to non-modified biochar, with experimental results showing that the adsorption efficiency of MBC quickly reached 99.02% for CV removal. Malika et al. (2023) [45] have shown that magnetized food-derived biochar had a better removal rate for Methyl orange and Congo red dye than biochar.

### 3.3. Adsorption Isotherm

**Figure 5:**
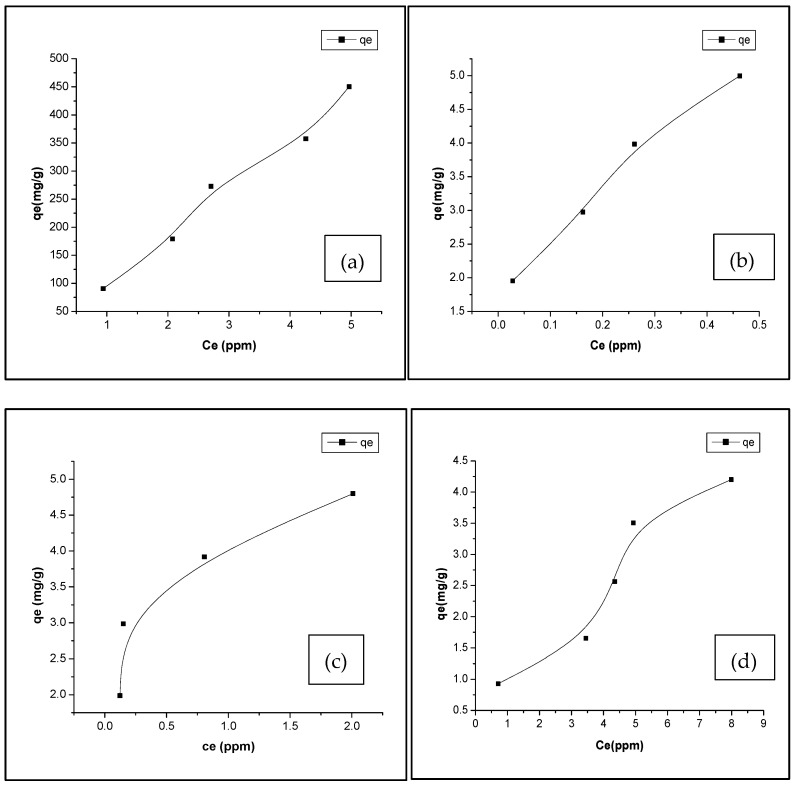
Adsorption isotherms of CV dye onto biochars at room temperature: (**a**) H_2_SO_4_-biochar, (**b**) Ag-biochar, (**c**) Fe-biochar, and (**d**) non-activated biochar.

The adsorption data were analyzed using both Langmuir and Freundlich isotherms following the practice of Anastasiou et al. (2024) and Shafiq et al. (2020) [46,47]. For a deeper insight into the adsorption characteristics, Table 3 presents the Langmuir and Freundlich isotherm parameters. The adsorption capacity (q_e_) was measured experimentally at different initial CV concentrations to observe adsorption trends. However, the true adsorption capacity of the biochar was derived from fitting the data to the Langmuir and Freundlich isotherm models, as presented in Table 3, with the Qe vs. Ce adsorption isotherm plotted in Figure 5 and the Freundlich isotherm plotted in Figure S1 (Supplementary material). This approach ensures that reported capacities reflect thermodynamic equilibrium behavior rather than concentration-specific measurements. The Langmuir isotherm model is based on the assumption of monolayer adsorption occurring on a surface containing a fixed number of uniform sites. The key parameters obtained from the Langmuir isotherm model are a correlation coefficient R^2^ of 0.44, a Langmuir constant *KL* of 0.12, a separation factor *RL* of 0.135, and a maximum adsorption capacity Qm of 7.73. The R^2^ value of 0.44 indicates a poor fit to the Langmuir isotherm, suggesting that this model does not adequately describe the adsorption process in this case. However, the separation factor *RL* of 0.135, which lies between 0 and 1, indicates that the adsorption is favorable. The positive value of *KL* (0.12) further supports the idea of favorable adsorption, although the relatively low value suggests a modest affinity between the adsorbate and the adsorbent.

The Freundlich isotherm, in contrast, is an empirical model representing adsorption on surfaces with varying site energies, providing key parameters including a correlation coefficient R^2^ of 0.89, a Freundlich constant *KF* of 1.05, and an adsorption intensity n of 1.59. The R^2^ value of 0.89 indicates a good fit to the Freundlich isotherm, suggesting that this model better describes the adsorption process compared to the Langmuir model. The Freundlich constant *KF* of 1.05 suggests a relatively high adsorption capacity, and the value of n is 1.59, which is greater than 1, indicating favorable adsorption and suggesting that the adsorption process is physical in nature.

A comparison of the two models indicates that the Freundlich isotherm provides a superior fit to the adsorption data, as reflected by its higher $R^{2}$ value (0.89) compared to that of the Langmuir isotherm (R^2^ = 0.44). Both models confirm that the adsorption process is favorable. Nevertheless, the greater correlation coefficient of the Freundlich model suggests it more accurately represents the adsorption behavior.

For the activated biochars, clear differences in adsorption performance were observed (Table 3). The H_2_SO_4_-activated biochar showed the highest adsorption capacity (*qm* = 488.2 mg/g), with excellent agreement to the Langmuir model (R^2^ = 0.982), confirming that acid activation substantially enhanced surface area and active sites. Fe-biochar also showed good adsorption behavior that fits most of the Freundlich model, suggesting heterogeneous adsorption mechanisms were more relevant. In contrast, Ag-biochar presented a relatively low adsorption capacity (*qm* = 5.56 mg/g), but the Freundlich model (R^2^ = 0.998) described its adsorption behavior very well, indicating strong surface heterogeneity despite the lower overall uptake. Taken together, these results confirm that surface activation strongly influences adsorption performance, with acid activation providing the greatest enhancement, while Freundlich fitting is generally a more reliable description of the adsorption process across all biochars.

However, the Langmuir model predicted a *qm* of 7.72 mg/g for the non-activated biochar, overestimating the experimental maximum adsorption capacity (4.20 mg/g) by 84%. For H_2_SO_4_-activated biochar, Langmuir *qm* (488.2 mg/g) slightly overestimated the experimental value (450.34 mg/g) by 8%. The Ag and Fe-modified biochars showed similar trends, with Langmuir predictions exceeding the experimental maxima by 11% and 10%, respectively. These differences can be attributed to the idealized assumptions of the Langmuir model, including monolayer adsorption on a homogeneous surface and uniform binding sites, which do not fully capture the heterogeneity and surface irregularities of the real biochars. Additionally, experimental factors such as incomplete equilibrium, pore diffusion limitations, or aggregation of modified biochars can lead to slightly lower observed qe values compared to model predictions. These differences are consistent with previous studies on heavy metal adsorption, where model-predicted capacities were slightly higher than experimental values due to assumptions of monolayer adsorption and uniform surface sites [47].

### 3.4. Soil Amendment and Irrigation

#### 3.4.1. Wheat Growth

**Figure 6:**
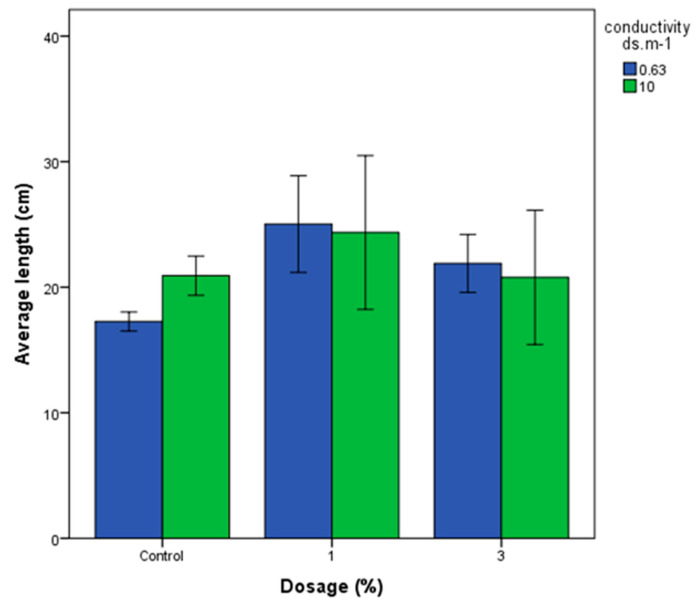
Effects of different biochar doses on the length and growth of wheat crops under varying water salinity conditions.

The effects of different biochar doses on the length and growth of wheat crops under varying water salinity conditions are presented in Figure 6. The results revealed that the highest recorded length was 26.6 cm, attributed to a 1% biochar application with a conductivity of 0.63 ds/m, while the lowest measured length was 17 cm in the control group with the same conductivity level. A low p-value of 0.001 for different biochar dosages indicates a statistically significant influence on wheat crop length and growth (Table 4 and Table 5. Further analysis indicates that the control group and the application of 3% biochar do not differ significantly. However, the 1% biochar application is markedly different from both the control group and the 3% biochar application, suggesting its favorable impact on wheat crop length and growth. In terms of water conductivity ranging from 0.63 ds/m to 10 ds/m, no significant impact (p-value: 0.495) was observed on crop length and growth. Thus, biochar application and dosage emerge as crucial factors that affect wheat crop length and growth, while variations in water conductivity within the specified range do not seem to have a notable effect on crop length.

#### 3.4.2. Chlorophyll Levels

**Figure 7:**
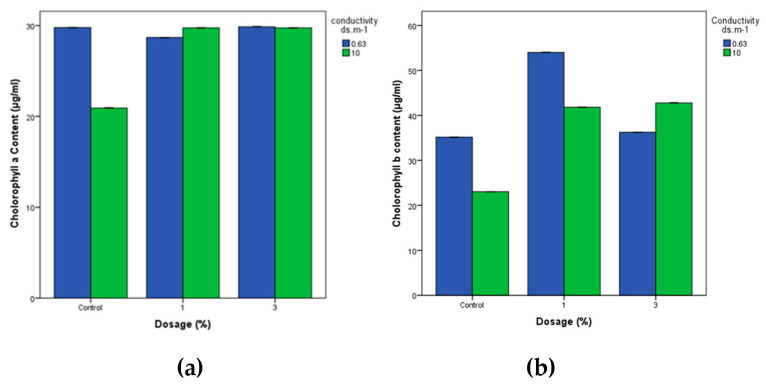
Effects of different biochar doses on the content of (**a**) chlorophyll a and (**b**) chlorophyll b in wheat under varying water salinity conditions.

Figure 7 shows the influence of different dosages of biochar on the chlorophyll a and b concentrations in wheat plants subjected to varying water salinity conditions. The results consistently showed higher chlorophyll levels in the biochar-treated groups compared to the control, indicating a positive impact of biochar on chlorophyll content. This suggests that biochar application may enhance plant health and photosynthetic activity, particularly under conditions of high salinity.

The measured chlorophyll levels ranged from 29.8 to 20.9 µg/mL for chlorophyll a and from 54 to 23 µg/mL for chlorophyll b. Statistical analysis revealed a significant effect of biochar dosage (*p* = 0.017) on chlorophyll a content in wheat plants. However, there were no significant differences between the 1% and 3% biochar dosage treatments in terms of chlorophyll a concentration. Additionally, the p-value of 0.043 associated with water salinity (conductivity) indicated a significant influence on chlorophyll a content in wheat plants. This underscores the importance of varying water salinity conditions (0.63 ds/m and 10 ds/m) in affecting chlorophyll levels in wheat plants (Table 6 and Table 7). In the absence of biochar, conductivity levels significantly affected chlorophyll levels, with both chlorophyll a and b content remarkably higher under irrigation with 0.63 ds/m compared to 10 ds/m (Table 6, Table 7, Table 8 and Table 9). This suggests that biochar may offer protection against the adverse effects of high water salinity on crops. Although the chlorophyll a content remained similar between the biochar dosage and control groups, the control group exhibited the lowest chlorophyll content at 10 ds/m. Under irrigation with 0.63 ds/m, the application of 1% biochar resulted in the highest chlorophyll concentration, surpassing the control group. Similarly, at 10 ds/m, the 1% biochar treatment showed the highest chlorophyll concentration, while the control group showed the lowest levels. In particular, overall chlorophyll concentrations were higher below 0.63 ds/m compared to 10 ds/m. Despite the higher salinity at 10 ds/m, biochar appeared to mitigate its adverse effects, indicating a protective role in preserving wheat crops under conditions of high salinity.

Therefore, in addition to the statistical analysis and in terms of practical significance, wheat seedlings treated with 1% biochar grew on average 25.3 cm compared to 17.3 cm in the control, representing an improvement of approximately 46%, while 3% biochar still produced a 25% increase in length. Under high salinity, 1% biochar increased shoot length from 21.0 cm in the control to 24.3 cm (16% increase), although 3% biochar showed little improvement. Similarly, chlorophyll concentrations were markedly enhanced by biochar. Chlorophyll b increased from 35 µg/mL in the control to 54 µg/mL (54% increase) with 1% biochar under low salinity, while under high salinity, the increase was even more pronounced from 23 to 43 µg/mL (87% increase). For chlorophyll a, no significant changes were observed under low salinity, but under high salinity, biochar boosted levels from 21 µg/mL in the control to 30 µg/mL (43% increase). These improvements are not only statistically reliable but also biologically meaningful, as longer shoots and higher chlorophyll content directly translate into stronger seedling establishment, enhanced photosynthetic activity, and greater resilience to salinity stress during early growth. Such physiological and morphological benefits align with previous reports indicating that biochar amendments enhance nutrient availability, water retention, and stress tolerance in crops grown under saline or degraded soils [34,48,49]. Importantly, our findings demonstrate that even at relatively low application rates (1–3%), biochar can exert significant positive effects, suggesting practical feasibility for field use in regions like the Beqaa Valley, where salinity constraints limit crop productivity, and where the Beqaa Valley is Lebanon’s main agricultural hub, and central to national wheat production and food security. However, increasing soil salinity and land degradation threaten its productivity, making amendments such as biochar vital for sustaining crop yields.

In examining irrigation practices and their influence on crop productivity, recent studies reveal the adverse effects of high electrical conductivity (EC) in irrigation water on wheat growth and yield. Mojid et al. (2014) [48] highlighted a significant decline in wheat growth and yield when EC levels in irrigation water exceed 10 dS·m^−1^, compared to lower EC levels (<1 dS·m^−1^). This sensitivity of wheat to elevated salinity emphasizes the critical impact of water quality on agricultural production. Tekin et al. (2014) [49] further delineated a salinity threshold for winter wheat, identifying 5.107 dS·m^−1^ as the EC level beyond which the reduction in wheat yield becomes apparent, based on two years of experimental data. Additionally, Wang et al. (2023) [50] identified that irrigation with water at an EC greater than 3.4 dS·m^−1^ negatively impacts wheat yield and leads to increased soil salt accumulation, underscoring the complex interplay between irrigation salinity and soil health. These studies collectively stress the need to manage irrigation water salinity to optimize wheat production, especially in regions where high-salinity irrigation sources are prevalent.

The potential of biochar to mitigate the negative impacts of saline irrigation water has also been substantiated through various studies, particularly in environments where salinity stress is a limiting factor. For instance, El Sayed et al. (2021) [51] conducted experiments in Egypt simulating saline groundwater conditions using drip irrigation. Their findings indicated that while saline water reduced grain yield, biochar application improved yield ratios, especially under high salinity. This highlights the role of biochar in alleviating saline stress, suggesting it as a viable amendment to improve wheat productivity in regions affected by salinity. Yerli (2023) [34] observed similar benefits of biochar, reporting a decrease in spinach growth parameters, such as plant height and chlorophyll content, with increasing salinity in irrigation water. However, the addition of biochar (at 3% by weight) improved these growth characteristics under saline conditions, indicating the potential of biochar to ameliorate salinity-induced stress.

Further evidence of biochar efficacy comes from studies that examine other crops and physiological markers. Helaoui et al. (2023) [52] noted increases in chlorophyll b levels when biochar was applied under saline conditions, while Vu et al. (2022) [53] and Sidra et al. (2017) [54] reported improvements in plant height, chlorophyll content, and shoot length with biochar under both saline and non-saline conditions. These findings suggest that biochar can enhance the resilience of crops to salinity, promoting better growth outcomes in stressful environments.

Collectively, these studies support biochar as a promising soil amendment to counteract the negative effects of irrigation salinity and enhance its potential role in sustainable agriculture. Consistent improvement in physiological and growth parameters with biochar application in different types of crops and saline conditions points to a broader applicability. Therefore, integrating biochar into soil management strategies could contribute to improved crop yields and resilience in salinity-affected agricultural systems, offering a sustainable approach to improve food security in saline-prone regions.

## 4. Conclusions

This study highlights the dual role of biochar in addressing water pollution and soil salinity, demonstrating its effectiveness as a sustainable solution for both wastewater treatment and agricultural resilience. Biochar derived from kitchen waste showed significant effectiveness in removing CV from contaminated water, with H_2_SO_4_-treated biochar achieving the highest adsorption capacity (450 mg/g) and consistently reaching a 100% removal rate at all tested concentrations. Silver-modified biochar also showed promising results, with a 99.95% removal efficiency at 50 ppm CV and additional potential for pathogen removal, suggesting future applications in water treatment beyond dye removal. Beyond water purification, biochar demonstrated its effectiveness in mitigating the negative effects of saline irrigation on wheat growth and yield. At a 1% application rate, biochar significantly improved wheat length, growth, and chlorophyll content, reducing the adverse impacts of high salinity and improving overall crop resilience. These findings underscore biochar’s potential as a cost-effective and eco-friendly amendment for improving soil conditions in saline environments.

Overall, this study demonstrated valuable understandings of biochar’s multifunctionality in environmental remediation and agricultural sustainability. Further research and implementation efforts can improve the applicability of biochar in addressing water contamination, including expanding the scope to cover wider experimental conditions such as concentration range and additional parameters such as temperature, contact time and soil degradation, contributing to improved crop productivity and long-term food security in regions affected by salinity stress.

## Figures and Tables

**Table 1:** XPS Elemental Composition and Peak Parameters of (a) Fe-Biochar, (b) Ag-Biochar, and (c) H_2_SO_4_-Biochar.

**(a)** **Name**	**Peak BE**	**FWHM eV**	**Area (P) CPS.eV**	**Atomic Percentage (%)**
C1s	284.84	1.63	4514.22	35.0
N1s	400.27	2.13	337.44	1.6
O1s	530.27	1.87	13,606.26	39.1
Na1s	1071.83	1.97	1824.19	1.9
Si2p	101.35	3.49	298.37	2.5
Fe2p	710.93	3.79	29,340.45	18.1
Cl2p	198.63	1.33	237.00	0.7
S2p	168.93	2.43	312.27	1.3
**(b)** **Name**	**Peak BE**	**FWHM eV**	**Area (P) CPS.eV**	**Atomic Percentage (%)**
C1s	284.84	1.73	7366.98	64.4
K2p	293.03	1.78	3863.72	7.2
Cl2p	198.68	2.78	395.72	1.2
P2s	190.16	2.18	96.76	0.7
O1s	531.47	2.56	6727.45	21.8
Na1s	1071.43	1.63	614.24	0.7
N1s	399.64	3.35	456.59	2.4
Ca2p	346.95	1.71	820.52	1.2
Ag3d5	367.82	1.23	227.36	0.2
**(c)** **Name**	**Peak BE**	**FWHM eV**	**Area (P) CPS.eV**	**Atomic Percentage (%)**
C1s	284.82	1.61	7941.62	69.6
S2p	168.77	2.35	655.13	3.0
N1s	401.04	2.60	589.25	3.2
O1s	532.12	3.04	7453.56	24.2

**Table 2:** Surface Area Analysis of Biochar and Its Modified Forms.

Sample	Surface Area (m^2^/g)
Biochar-H_2_SO_4_	33.0
Biochar-Ag	3.4
Biochar	4.8
Biochar-Fe	4.76

**Table 3:** Langmuir and Freundlich isotherm parameters of non-activated biochar and of activated biochar.

Biochar	Model	*qm* (mg/g)	*KL*	*RL*	*R* ^2^	*KF*	*n*
Non-activated	Langmuir	7.72	0.12	0.13	0.44	–	–
	Freundlich	–	–	–	0.89	1.05	1.59
H_2_SO_4_-biochar	Langmuir	488.20	0.02	0.50	0.982	–	–
	Freundlich	–	–	–	0.97	97.07	1.06
Ag-biochar	Langmuir	5.56	17.99	0.001	0.44	–	–
	Freundlich	–	–	–	0.998	5.5	3.57
Fe-biochar	Langmuir	5.26	7.20	0.028	0.909	–	–
	Freundlich	–	–	–	0.912	4.078	4.06

**Table 4:** ANOVA summary for dosage and conductivity effects on wheat crop length.

Source	Type III Sum of Squares	df	Mean Square	F	Sig.
Corrected Model	97.1601	3	32.387	9.121	0.001
Intercept	8482.748	1	8482.748	2388.904	0.000
Dosage	95.420	2	47.710	13.436	0.001
conductivity	1.739	1	1.739	0.490	0.495
Error	49.713	14	3.551		
Total	8629.620	18			
Corrected Total	146.872	17			

**Table 5:** Comparisons of biochar dosage levels on wheat crop length.

Dosage (I)	Dosage (J)	Mean Difference (I-J)	Std. Error	Sig.	95% Confidence Interval	
					Lower Bound	Upper Bound
0	1%	−5.603 *	1.069	0.000	−8.48	−2.72
	3%	−2.248	1.069	0.159	−5.13	0.63
1%	0	5.603 *	1.069	0.000	2.72	8.48
	3%	3.356 *	1.069	0.020	0.48	6.24
3%	0	2.248	1.069	0.159	−0.63	5.13
	1%	−3.356 *	1.069	0.020	−6.24	−0.48

* The mean difference is significant at the 0.05 level.

**Table 6:** ANOVA summary of the effects of biochar dosage and conductivity on the chlorophyll a content in wheat leaves.

Source	Type III Sum of Squares	df	Mean Square	F	Sig.
Corrected Model	101.555 ^a^	3	33.852	5.379	0.011
Intercept	14,243.979	1	14,243.979	2263.266	0.000
dosage	70.240	2	35.120	5.580	0.017
conductivity	31.314	1	31.314	4.976	0.043
Error	88.110	14	6.294		
Total	14,433.643	18			
Corrected Total	189.664	17			

^a^ R Squared = 0.535 (Adjusted R Squared = 0.436).

**Table 7:** Comparisons of biochar dosage levels on chlorophyll a content.

(I) Dosage	(J) Dosage	Mean Difference (I-J)	Std. Error	Sig. ^b^	95% Confidence Interval for Difference ^b^	
					Lower Bound	Upper Bound
0	1	−3.860 *	1.448	0.018	−6.966	−0.753
	3	−4.457 *	1.448	0.008	−7.564	−1.351
1	0	3.860 *	1.448	0.018	0.753	6.966
	3	−0.597	1.448	0.686	−3.704	2.509
3	0	4.457 *	1.448	0.008	1.351	7.564
	1	0.597	1.448	0.686	−2.509	3.704

* The mean difference is significant at the 0.05 level. ^b^ Adjustment for multiple comparisons: Least Significant Difference (equivalent to no adjustments).

**Table 8:** ANOVA summary for the effects of biochar dosage and conductivity on chlorophyll b content in wheat leaves.

Source	Type III Sum of Squares	df	Mean Square	F	Sig.
Corrected Model	915.342 ^a^	3	305.114	15.248	0.000
Intercept	17,393.915	1	17,393.915	869.246	0.000
Dosage	817.234	2	408.617	20.420	0.000
Conductivity	98.108	1	98.108	4.903	0.044
Error	280.145	14	20.010		
Total	18,589.402	18			
Corrected Total	1195.487	17			

^a^ R Squared = 0.766 (Adjusted R Squared = 0.715).

**Table 9:** Comparisons of biochar dosage levels on chlorophyll b content of wheat crops.

(I) Dosage	(J) Dosage	Mean Difference (I-J)	Std. Error	Sig. ^b^	95% Confidence Interval for Difference ^b^	
					Lower Bound	Upper Bound
0	1	−16.505 *	2.583	0.000	−22.044	−10.966
	3	−8.281 *	2.583	0.006	−13.820	−2.741
1	0	16.505 *	2.583	0.000	10.966	22.044
	3	8.224 *	2.583	0.007	2.685	13.763
3	0	8.281 *	2.583	0.006	2.741	13.820
	1	−8.224 *	2.583	0.007	−13.763	−2.685

Based on estimated marginal means. * The mean difference is significant at the 0.05 level. ^b^ Adjustment for multiple comparisons: Least Significant Difference (equivalent to no adjustments).

## Data Availability

The original contributions presented in this study are included in the article/supplementary material. Further inquiries can be directed to the corresponding authors.

## Supplementary Materials

The following supporting information can be downloaded at: https://www.mdpi.com/article/10.3390/ma18204678/s1, Figure S1. Freundlich isotherm for adsorption of CV on biochar at room temperature: (a) Ag-biochar, (b) Fe-biochar, (c) non-activated biochar, and (d) H_2_SO_4_-biochar.
