# Supplementary material for: Evaluating Biochar’s Role in Dye Adsorption and Wheat Performance Under Saline Conditions
Source: Materials (Basel). 2025 Oct 12;18(20):4678. doi: 10.3390/ma18204678 (PMC12565813; doi:10.3390/ma18204678)
Supplement: Supplementary file 1 [file materials-18-04678-s001.zip › materials-3871646-supplementary.pdf]

Evaluating Biochar's Role in Dye Adsorption and Wheat Performance Under Saline Conditions

Ghenwa Kataya [1,2], Dalia El Badan [3,4], David Cornu [2], Assi Al Mousawi [5], Mikhael Bechelany [2,*] and Akram Hijazi [1]

[1] Research Platform for Environmental Science (PRASE),Doctoral School of Science and Technology, Lebanese University, Hadath 1519, Lebanon; ghenwa.kataya.1@ul.edu.lb (G.K.); akram.hijazi@ul.edu.lb (A.H.)

[2] Institut Européen des Membranes, IEM–UMR 5635, University of Montpellier, CNRS, ENSCM, Place Eugène Bataillon, 34095 Montpellier, France; david.cornu@umontpellier.fr

[3] Department of Biological Sciences, Faculty of Science, Beirut Arab University, Beirut P.O. Box 11-5020, Leba non; d.badan@bau.edu.lb

[4] Botany and Microbiology Department, Faculty of Science, Alexandria University, Alexandria 21568, Egypt

[5] Department of Biological and Chemical Sciences, School of Arts and Sciences, Lebanese International University, Khiyara—West Bekaa, Beirut P.O. Box 146404, Lebanon; assi.mousawi@liu.edu.lb

* Correspondence: mikhael.bechelany@umontpellier.fr

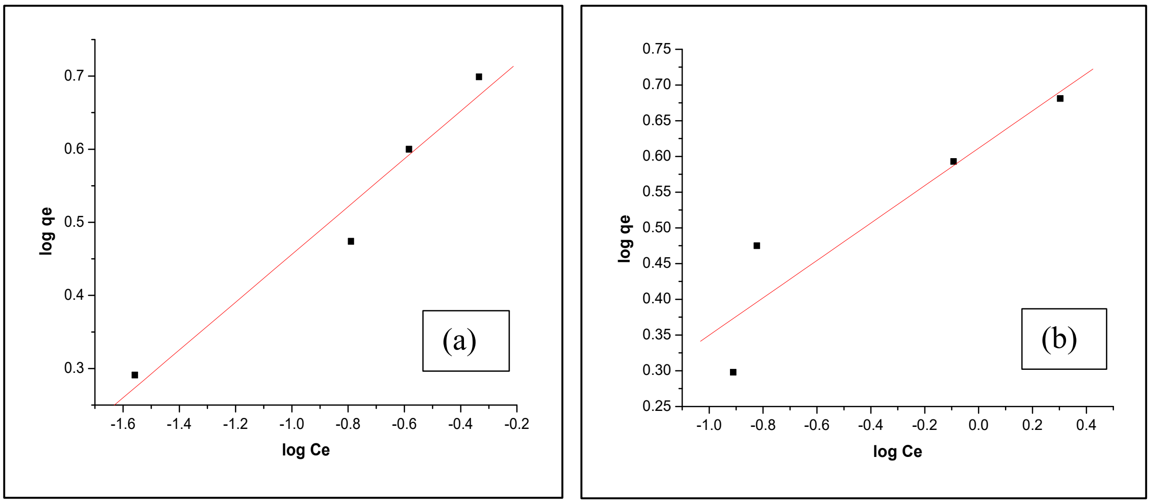

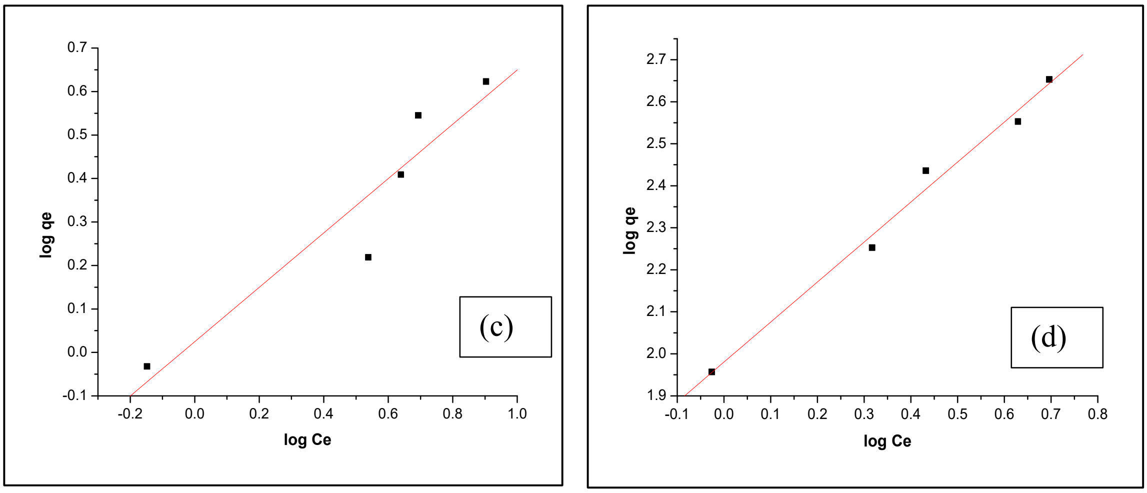

Figure S1. Freundlich isotherm for adsorption of CV on biochar at room temperature: (a) Ag-biochar, (b) Fe-biochar, (c) non-activated biochar, and (d) H_2SO_4-biochar.
